# Supplementary material for: Application of referenced thermodynamic integration to Bayesian model selection
Source: PLoS One. 2023 Aug 14;18(8):e0289889. doi: 10.1371/journal.pone.0289889 (PMC10424863; doi:10.1371/journal.pone.0289889)
Supplement: S1 Appendix — (PDF) [file pone.0289889.s001.pdf]

Application of Referenced Thermodynamic Integration to Bayesian Model Selection

SI 1 - Variational Laplace Reference

The conditions to identify an optimal reference normalising constant can be derived by considering a Taylor expansion of the log normalising constant $\log z(\lambda)$ about $\lambda = 0$:

$$\log z(\lambda) \approx \log z(0) + \lambda\, \partial_\lambda \log z(0) + \frac{1}{2}\lambda^2\, \partial^2_\lambda \log z(0)\,.$$

The first derivative gives the expectation

$$\partial_\lambda \log z(\lambda) = \mathbb{E}_{q(\lambda;\boldsymbol{\theta})}\left[\log \frac{q(\boldsymbol{\theta})}{q_{\text{ref}}(\boldsymbol{\theta})}\right],$$

as per the derivation in Eq 2, and the second derivative is a variance

$$\begin{aligned}
\partial^2_\lambda \log z(\lambda) &= \frac{\int \left(\log \frac{q(\boldsymbol{\theta})}{q_{\text{ref}}(\boldsymbol{\theta})}\right)^2 q(\lambda;\boldsymbol{\theta}) d\boldsymbol{\theta}}{\int q(\lambda;\boldsymbol{\theta}) d\boldsymbol{\theta}} - \left(\frac{\int \left(\log \frac{q(\boldsymbol{\theta})}{q_{\text{ref}}(\boldsymbol{\theta})}\right) q(\lambda;\boldsymbol{\theta}) d\boldsymbol{\theta}}{\int q(\lambda;\boldsymbol{\theta}) d\boldsymbol{\theta}}\right)^2 \\
&= \left\{\mathbb{E}_{q(\lambda;\boldsymbol{\theta})}\left[\left(\log \frac{q(\boldsymbol{\theta})}{q_{\text{ref}}(\boldsymbol{\theta})}\right)^2\right] - \mathbb{E}_{q(\lambda;\boldsymbol{\theta})}\left[\log \frac{q(\boldsymbol{\theta})}{q_{\text{ref}}(\boldsymbol{\theta})}\right]^2\right\} \\
&\geq 0\,.
\end{aligned}$$

As the curvature of $\log z(\lambda)$ is increasing, to first order we see

$$\log z(\lambda) \geq \log z(0) + \lambda \mathbb{E}_{q(0;\boldsymbol{\theta})}\left[\log \frac{q(\boldsymbol{\theta})}{q_0(\boldsymbol{\theta})}\right],$$

and for the specific case of $\lambda = 1$,

$$\log z \geq \log z_{\text{ref}} + \mathbb{E}_{q_{\text{ref}}(\boldsymbol{\theta})}\left[\log \frac{q(\boldsymbol{\theta})}{q_{\text{ref}}(\boldsymbol{\theta})}\right].$$

This inequality establishes bounds that can be maximised with respect to the position ($\boldsymbol{\mu}$) and scale ($\boldsymbol{S}$) parameters of a reference density such as

$$q_{\text{ref}}(\boldsymbol{\mu}, \boldsymbol{S}; \boldsymbol{\theta}) = q(\boldsymbol{\mu}) \exp\left(-\frac{1}{2}(\boldsymbol{\theta} - \boldsymbol{\mu})^{\mathrm{T}} \boldsymbol{S}\,(\boldsymbol{\theta} - \boldsymbol{\mu})\right).$$

Thus the parameters that optimise

$$\max_{\boldsymbol{\mu}, \boldsymbol{S}} \left\{\log z_{\text{ref}} + \mathbb{E}_{q_{\text{ref}}(\boldsymbol{\theta})}\left[\log \frac{q(\boldsymbol{\theta})}{q_{\text{ref}}(\boldsymbol{\mu}, \boldsymbol{S}; \boldsymbol{\theta})}\right]\right\},$$

provide a reference density that is variationally optimal. We note this is an application of the Gibbs-Feynman-Bogoliubov inequality [1–3], and that finding approximations of this type to the true density is a well-studied problem in machine learning, with well-documented approaches that can be used to determine q_{ref} variationally [4, 5]. In itself the existence of a variational bound provides no guarantee of being a good approximation to the true normalising constant, and is thus alone not a satisfactory general approach. However as a point of reference from which to estimate the true normalising constant, it provides a first-order optimal density within the family of trial reference functions considered, therefore improving convergence to the MCMC normalising constant in referenced TI.

References

1. Bogolubov Jr NN. On model dynamical systems in statistical mechanics. Physica. 1966;32(5):933–944.

2. Kuzemsky AL. Variational principle of Bogoliubov and generalized mean fields in many-particle interacting systems. International Journal of Modern Physics B. 2015;29(18):1530010.

3. Zhang J. The application of the Gibbs-Bogoliubov-Feynman inequality in mean field calculations for Markov random fields. IEEE Transactions on Image Processing. 1996;5(7):1208–1214.

4. Neal RM, Hinton GE. A view of the EM algorithm that justifies incremental, sparse, and other variants. In: Learning in graphical models. Springer; 1998. p. 355–368.

5. Jordan MI, Ghahramani Z, Jaakkola TS, Saul LK. An introduction to variational methods for graphical models. Machine learning. 1999;37(2):183–233.
