# Supplementary material for: Application of referenced thermodynamic integration to Bayesian model selection
Source: PLoS One. 2023 Aug 14;18(8):e0289889. doi: 10.1371/journal.pone.0289889 (PMC10424863; doi:10.1371/journal.pone.0289889)
Supplement: S2 Appendix — (PDF) [file pone.0289889.s002.pdf]

Application of Referenced Thermodynamic Integration to Bayesian Model Selection

SI 2 - Applications

Here we present additional figures and tables for the examples shown in Section Applications of the paper.

2D Pedagogical Example with Constrained Parameters

Table 1. Evidence calculated with different methods. Constraint correction refers to imposing the integration limits on the reference as per Eq 8 in the main text. Diagonal covariance means a covariance matrix where only the diagonal (variance) terms are non-zero. Full covariance is a covariance matrix in which all terms can be non-zero. * obtained numerically [1, 2].

Method	Evidence
Exact*	3.31
Laplace with full covariance	5.55
Laplace with diagonal covariance + constraint correction	3.81
Ref TI with full covariance	4.79
Ref TI with diagonal covariance + constraint correction	3.33

Benchmarks – *Radiata Pine*

Table 2. Comparison of Bayes factors for *radiata pine* models for each method. Here we show $BF_{21} = \frac{M_2}{M_1}$ to determine whether model M_2 is better than model M_1. Both TI and referenced TI methods used 11 equidistant λ-s. Power posteriors method was used with 11 (PP_{11}) and 100 (PP_{100}) λ-s. Third column shows the total number of MCMC steps required to achieve standard error of 0.5%, excluding the warm-up steps. * - using sampled covariance matrix.

Method	BF_{21}	MCMC steps
Exact	4552.35	-
Laplace approximation*	6309.10	-
Model switch TI	4557.63	2,365
Referenced TI	4558.71	308
PP_{11}	4463.71	41,514
PP_{100}	4757.82	55,000

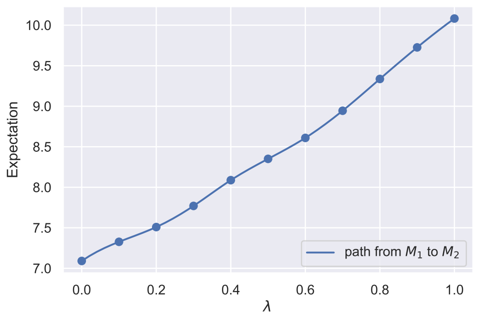

(a) Model switch TI

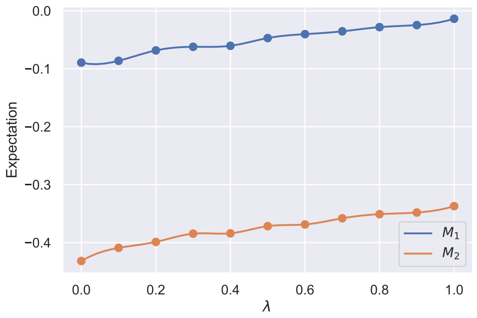

(b) Referenced TI

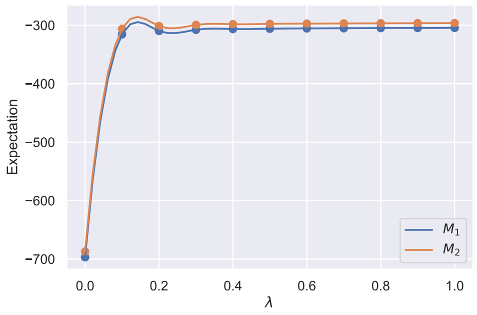

(c) PP_{11}

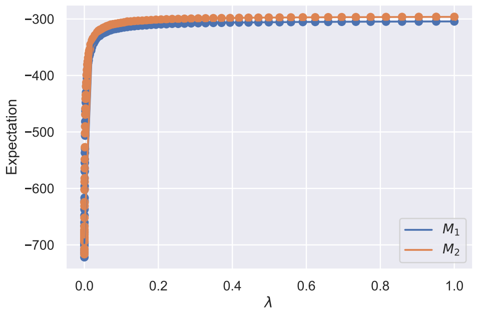

(d) PP_{100}

Fig 1. The HMC-evaluated expectation of $\mathbb{E}_{q(\lambda;\boldsymbol{\theta})}\left[\log \frac{q(\boldsymbol{\theta})}{q_{\text{ref}}(\boldsymbol{\theta})}\right]$ vs coupling parameter λ is given for models M_1 and M_2 for four methods of calculating the model evidence: model switch TI (a), referenced TI (b), power posteriors with 11 λ-placements (c) and power posteriors with 100 λ placements (d). Model switch TI (a) creates the path directly between two competing densities, therefore only one line is shown (see Eq 2 in the main text). In each of the plots, the evaluated expectation for a given λ is shown with the dot, and the lines connecting the dots were obtained through interpolation.

COVID-19 Model

The COVID-19 model shown is based on the renewal equation derived from the Bellman-Harris process. The details of the model and its derivation are provided in [3]. Here, we give a short overview of the $AR(2)$ model. The model has a Bayesian hierarchical structure and is fitted to the time-series data containing a number of new confirmed COVID-19 cases per day in South Korea from 31-12-2019 to 18-07-2020, obtained from `https://opendata.ecdc.europa.eu/covid19/casedistribution/csv`. New infections $y(t)$ are modelled by a negative binomial distribution, with a mean parameter in a form of a renewal equation. The number of confirmed cases $y(t)$ is modelled as:

$$y \sim \text{NegBin}(f(t), \phi)\,,$$

where ϕ is an overdispersion or variance parameter and the mean of the negative binomial distribution is denoted as $f(t)$ and represents the daily case data through:

$$f(t) = R_t \sum_{\tau<t} f(t-\tau)g(\tau)\,.$$

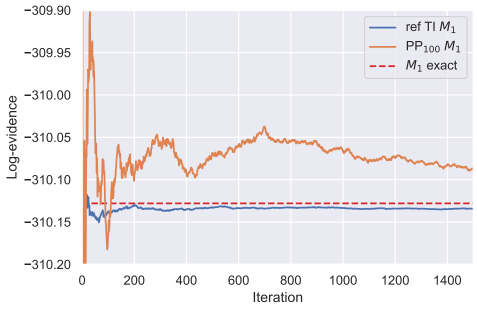

(a) M_1

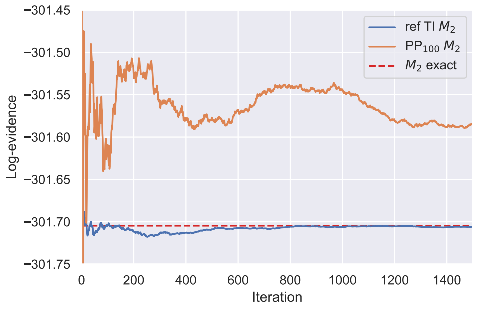

(b) M_2

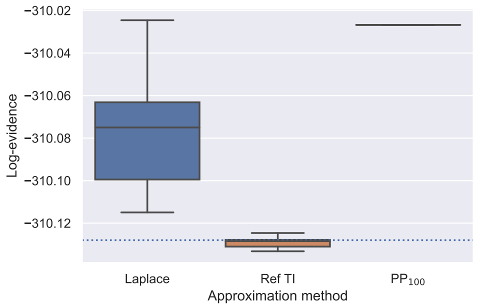

(c) M_1

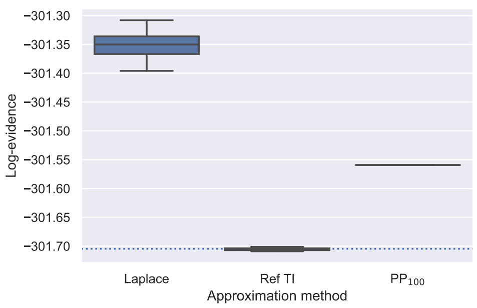

(d) M_2

Fig 2. Log-evidence of M_1 and M_2 for the three algorithms. (a) and (b) show the rolling mean of log-evidence of M_1 and M_2 over 1500 iterations per λ obtained by referenced TI (blue line) and PP$_{100}$ (orange line) methods. The exact value is shown with red dashed line. (c) and (d) show the mean log-evidence of the two models evaluated over 15 runs of the three algorithms. The exact value of the log-evidence is shown with the dotted line.

Here, $g(\tau)$ is a Raleigh-distributed serial interval with mean GI, which is discretised as

$$g_s = \int_{s-0.5}^{s+0.5} g(\tau)d\tau \text{ for } s = 2, 3, ... \text{ and } g_1 = \int_{0}^{1.5} g(\tau)d\tau \,.$$

R_t, the effective reproduction number, is parametrised as $R_t = \exp(\epsilon_t)$, with exponent ensuring positivity. ϵ_t is an autoregressive process with two-days lag, that is AR(2), with $\epsilon_1 \sim N(-1, 0.1)$, $\epsilon_2 \sim N(-1, \sigma)$ and

$$\epsilon_t \sim N(\rho_1 \epsilon_{t-1} + \rho_2 \epsilon_{t-2}, \sigma_t) \text{ for } t = \{3, 4, 5, ...\}.$$

The model's priors are:

$$\begin{aligned}
\sigma &\sim N^{+}(0, 0.2)\,,\\
\rho_1 &\sim N^{+}(0.8, 0.05)\,,\\
\rho_2 &\sim N^{+}(0.1, 0.05)\,,\\
\phi &\sim N^{+}(0, 5)\,,\\
GI &\sim N^{+}(0.01, 001)\,.
\end{aligned}$$

Modification were applied to this basic model, to obtain the different variants of the model as described in Section Applications. First group of models analysed was the

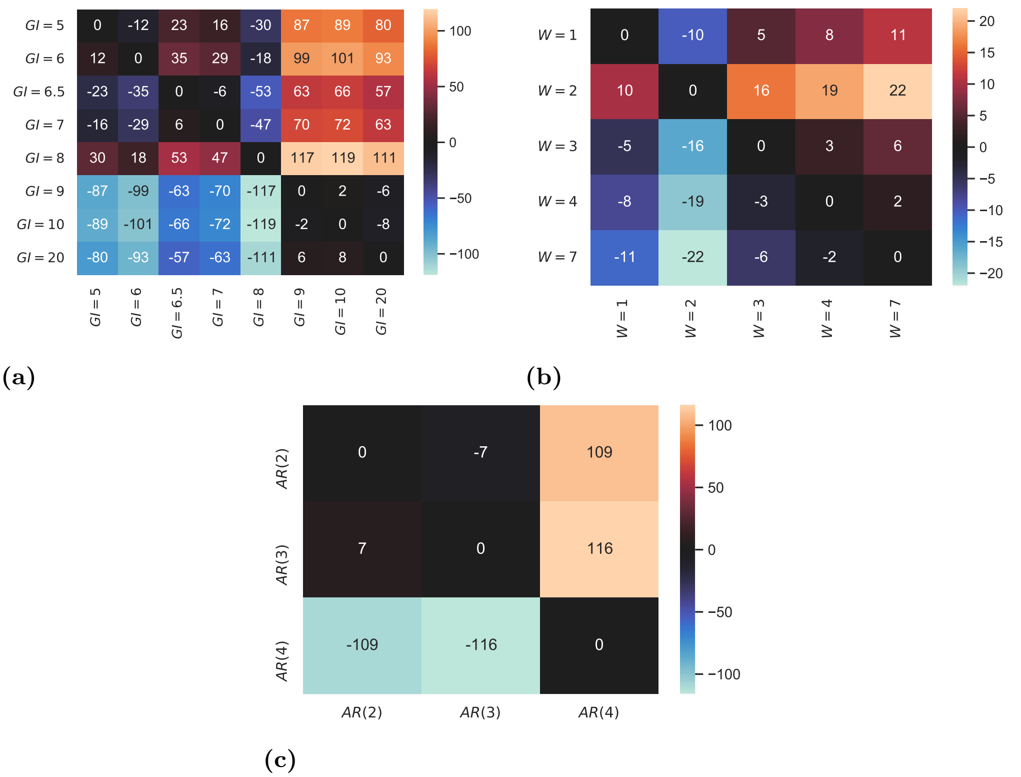

Fig 3. Logarithms of Bayes factors for the analysed COVID-19 renewal models, evaluated using the normalising constants ratios obtained by referenced TI. In each cell, the colour indicates the value of the $BF_{1,2}$ for models M_1 (row) and M_2 (column). Higher values, that is a brighter orange colour, suggest that M_1 is strongly better than M_2, and values below 0 in blue palette indicate that M_1 is worse than M_2. $GI = 8$ performed best out of fixed GI models, $W = 2$ best out of sliding window models, and $AR(3)$ performed better than $AR(2)$ and $AR(4)$. For the interpretation of the BF values see [4].

AR(2) model described above, but with the GI parameter fixed to a certain value instead of inferring that parameter from the data. $AR(3)$ and $AR(4)$ models had additional parameters ρ_3 and ρ_4, which allow to model the autoregressive process with a longer lag (3- and 4- days respectively). Finally, models $W = k$, $k = 1, .., 7$ were similar to the $AR(2)$ model, but the underlying assumption of these models is that the R_t stays constant for the duration of the length of the sliding window $W = k$.

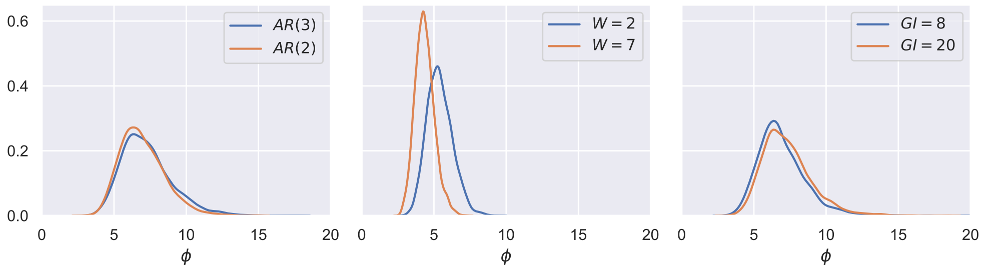

(a) Posterior distributions for overdispersion parameter ϕ

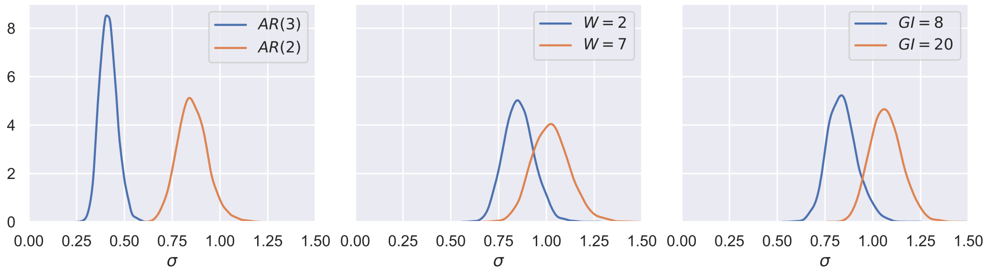

(b) Posterior distributions for σ parameter

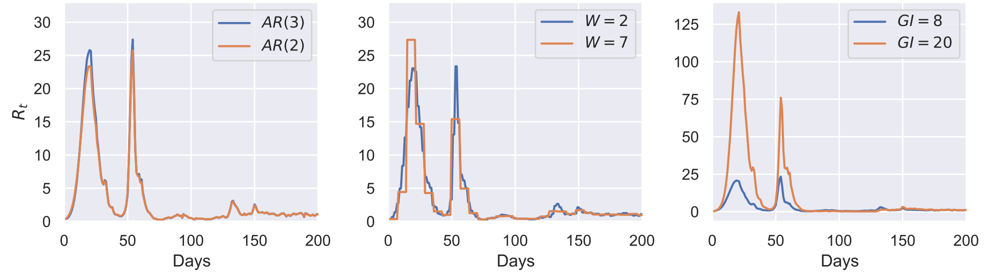

(c) R_t generated by the favoured models

Fig 4. Posterior distributions for models' parameters for models favoured by BFs using the Laplace approximation (orange lines) and referenced TI (blue lines).

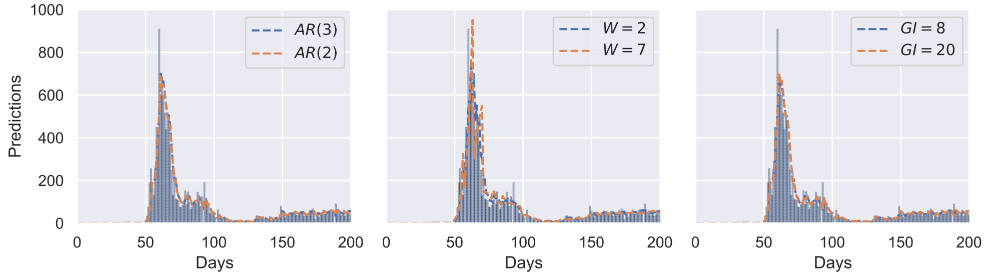

Fig 5. Cases of SARS-CoV-2 infections in South Korea from the data (shown with bars) and the cases predicted by different models. On each graph, predictions made by the model favoured by the Laplace approximation is shown with a blue dashed line, and predictions made by the referenced TI favoured models are shown with an orange dashed line. The lines in all three subplots are largely overlapping, revealing that all models fitted the case data similarly well.

References

1. Virtanen P, Gommers R, Oliphant TE, Haberland M, Reddy T, Cournapeau D, et al. SciPy 1.0: Fundamental Algorithms for Scientific Computing in Python. Nature Methods. 2020;17:261–272. doi:10.1038/s41592-019-0686-2.

2. Piessens R, deDoncker Kapenga E, Ueberhuber C, Kahaner D. QUADPACK: A Subroutine Package for Automatic Integration. Springer; 1983.

3. Berah T, Mellan TA, Miscouridou X, Mishra S, Parag KV, Pakkanen MS, et al. Unifying the effective reproduction number, incidence, and prevalence under a stochastic age-dependent branching process. arXiv preprint. 2021;doi:10.48550/arXiv.2107.05579.

4. Kass RE, Raftery AE. Bayes Factors. Journal of the American Statistical Association. 1995;90(430):773–795.
