# Supplementary material for: Application of referenced thermodynamic integration to Bayesian model selection
Source: PLoS One. 2023 Aug 14;18(8):e0289889. doi: 10.1371/journal.pone.0289889 (PMC10424863; doi:10.1371/journal.pone.0289889)
Supplement: S3 Appendix — (PDF) [file pone.0289889.s003.pdf]

Application of Referenced Thermodynamic Integration to Bayesian Model Selection

SI 3 - Bias and variance

Although referenced thermodynamic integration and other methods using path-sampling have theoretical asymptotically exact Monte Carlo estimator limits, in practice a number of considerations affect accuracy. For example, biases will be introduced to the referenced TI estimate in practice if one endpoint density substantially differs from another. Then the volume of parameter space that must be explored to produce an unbiased estimate of the expectation cannot be sampled based on the reference density generating proposals within a practical number of iterations. The point is shown for a simple 1D example in Fig 1. Similarly, the larger the mismatch, the higher the variance and slower the expectation is to converge. This illustrates the advantage of using a reference that matches the posterior as closely as possible, as opposed to a typically wide reference like the prior distribution, that gives the characteristic divergence at $\lambda = 0$ with power posteriors. Measures of density similarity in path sampling have been discussed by [1], however in practical terms there remains much scope for analysis of reference performance in terms of scaling with distribution dimension and type, which should be considered in detail in future work.

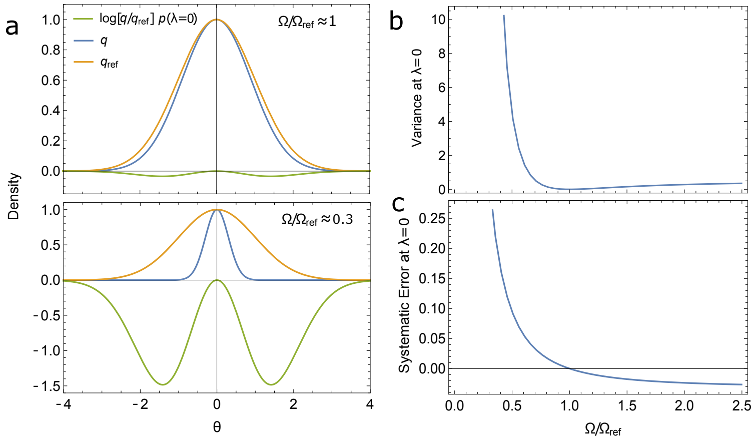

Fig 1. a) 1D examples to illustrate the bias and variance introduced with finite MCMC samples when q and q_{ref} are mismatched. In these examples Ω and Ω_{ref} denote the domain of the 99% quartiles of q and q_{ref}. b) A mismatch between q and q_{ref} (Ω and Ω_{ref}) causes the variance of $\log\frac{q}{q_{\text{ref}}}$ to increase, requiring more iterations to convergence. c) Similarly the mismatch causes the mass of the distribution for the expectation of $\log\frac{q}{q_{\text{ref}}}$ (evaluated with respect to the reference distribution) to increase beyond the parameter range effectively sampled with finite iterations, in this example corresponding to the 99% quartile of the sampling distribution, thus introducing a bias in the expectation.

References

1. Lefebvre G, Steele R, Vandal AC. A path sampling identity for computing the Kullback–Leibler and J divergences. Computational Statistics & Data Analysis. 2010;54(7):1719–1731. doi:10.1016/j.csda.2010.01.018.
